# An aldehyde dehydrogenase gene, *GhALDH7B4_A06*, positively regulates fiber strength in upland cotton (*Gossypium hirsutum* L.)

**DOI:** 10.3389/fpls.2024.1377682

**Published:** 2024-04-26

**Authors:** Liyuan Tang, Cunjing Liu, Xinghe Li, Haitao Wang, Sujun Zhang, Xiao Cai, Jianhong Zhang

**Affiliations:** Institute of Cotton, Hebei Academy of Agriculture and Forestry Sciences, Key Laboratory of Cotton Biology and Genetic Breeding in Huanghuaihai Semiarid Area, Ministry of Agriculture and Rural Affairs, Shijiazhuang, Hebei, China

**Keywords:** fiber strength, bulk segregant analysis (BSA), quantitative trait locus (QTL), *GhALDH7B4_A06*, upland cotton (*Gossypium hirsutum* L.)

## Abstract

High fiber strength (FS) premium cotton has significant market demand. Consequently, enhancing FS is a major objective in breeding quality cotton. However, there is a notable lack of known functionally applicable genes that can be targeted for breeding. To address this issue, our study used specific length–amplified fragment sequencing combined with bulk segregant analysis to study FS trait in an F_2_ population. Subsequently, we integrated these results with previous quantitative trait locus mapping results regarding fiber quality, which used simple sequence repeat markers in F_2_, F_2:3_, and recombinant inbred line populations. We identified a stable quantitative trait locus *qFS_A06_* associated with FS located on chromosome A06 (90.74–90.83 Mb). Within this interval, we cloned a gene, *GhALDH7B4_A06*, which harbored a critical mutation site in coding sequences that is distinct in the two parents of the tested cotton line. In the paternal parent Ji228, the gene is normal and referred to as *GhALDH7B4_A06^O^*; however, there is a nonsense mutation in the maternal parent Ji567 that results in premature termination of protein translation, and this gene is designated as truncated *GhALDH7B4_A06^S^*. Validation using recombinant inbred lines and gene expression analysis revealed that this mutation site is correlated with cotton FS. Virus-induced gene silencing of *GhALDH7B4* in cotton caused significant decreases in FS and fiber micronaire. Conversely, *GhALDH7B4_A06^O^* overexpression in *Arabidopsis* boosted cell wall component contents in the stem. The findings of our study provide a candidate gene for improving cotton fiber quality through molecular breeding.

## Introduction

Cotton is a primary natural fiber source in the textile industry (Wen et al., 2023). Upland cotton (*Gossypium hirsutum* L.) is extensively cultivated because of its high yield and adaptability, and it accounts for over 95% of cultivated cotton (Yuan et al., 2019). Because of the negative correlation between cotton yield and fiber quality, relying on traditional breeding to simultaneously improve yield and quality is challenging (Wang et al., 2020). This has resulted in moderate fiber quality of commonly cultivated varieties. With the improvement of modern textile processes, the growing consumer demand for high-quality cotton products, and damage to cotton fibers caused by current mechanical harvesting, there is a substantial market demand for premium cotton with high fiber strength (FS) (Zang et al., 2022). Enhancing FS has become a major goal in cotton quality breeding.

Cotton fiber, a single-cell trichome with an extended and thickened seed surface, also serves as an excellent model for investigating cellular development processes (Haigler et al., 2012; Xu et al., 2021). Cotton fiber development progresses through four interconnected stages: fiber initiation, elongation, secondary cell wall (SCW) thickening, and desiccation maturation. Fiber length (FL; mm), fiber strength (FS; cN/tex), fiber uniformity ratio (FU), fiber micronaire (FM), and fiber elongation (FE) are the primary variables regulating the fiber’s characteristics (Liu et al., 2016). FS primarily develops during the SCW thickening stage (Zang et al., 2022). SCW thickening is an intricate biological process that is controlled by the biosynthesis-related genes responsible for cell wall primary components, cellulose, hemicellulose, and lignin, and it is regulated by numerous transcription factors, microRNAs, and phytohormones (Kumar and Turner, 2015; Zang et al., 2022; Ma et al., 2018; Huang et al., 2018; Li et al., 2018; Cao et al., 2020; Sun et al., 2020; Zhang et al., 2021; Tang et al., 2023). Furthermore, it is apparent that there are numerous genes and complex regulatory networks associated with SCW. For example, among NAC (NAM, ATAF and CUC) type transcription factors, a total of 38 *GhNAC* genes have been identified to be involved in cotton fiber development (Sun et al., 2018). FS value is influenced by cellulose deposition, which determines cell wall thickness, and cellulose fiber organization, which determines cellulose crystallinity, during the critical period of SCW thickening (Zang et al., 2022).

Previous researchers revealed numerous qualitative trait loci (QTLs) related to FS by employing linkage analysis or genome-wide association study using various markers such as amplified fragment length polymorphisms, restriction fragment length polymorphisms, and simple sequence repeats (SSRs) on samples from different populations (Fang et al., 2017; Ning et al., 2014). With the refinement of cotton reference genome sequencing (Li et al., 2015; Zhang et al., 2015; Hu et al., 2019; Wang et al., 2019; Ma et al., 2021), high-density single-nucleotide polymorphisms (SNPs), individually and in conjunction with SSRs and other markers, were used to identify several FS-related genes (Islam et al., 2016; Li et al., 2016; Sun et al., 2017; Zhang et al., 2017; Ma et al., 2018; Feng et al., 2020; He et al., 2021; Yang et al., 2022). For example, Ma et al. (2018) conducted a re-sequencing study on 419 core germplasm resources and detected 630 FS-related SNPs. Keerio et al. (2018) mapped six FS-related QTLs on three chromosomes using specific length–amplified fragment sequencing (SLAF-seq) of an introgression line population and its parent. These studies identified FS-related SNPs and genes, which increased the density of marker loci in intraspecific genetic maps and greatly improved candidate gene location accuracy.

Numerous FS-related candidate genes have been identified and the functions of some genes validated (Zang et al., 2021). Nonetheless, many potential genes have yet to be confirmed, and this is largely attributed to genotypic limitations and protracted transformation periods involved in establishing stable transgenic cotton plants with targeted characteristics via genetic modification (Ge et al., 2023). Virus-induced gene silencing (VIGS) is a rapid method commonly employed for preliminary gene function determination (Tian et al., 2022). VIGS studies have revealed the role of strigolactone biosynthetic genes and strigolactone-responsive transcription factor genes in modulating cotton fiber development because they affect SCW thickness and fiber elongation (Tian et al., 2022; Wen et al., 2023). Other research has used VIGS to demonstrate the involvement of genes such as *GhERF41*, *GhLTP1*, *GhSTLs*, and *GhAPs* in the formation of FL or FS, respectively (Deng et al., 2016; Guo et al., 2022; Gao et al., 2023; Zhang et al., 2024). Similarly, *Arabidopsis* serves as a useful model for fiber trait investigations, with studies showing that overexpression of genes such as *GhMYB7*, *GhMYB25*-like, and *GhCesAs* can modify cell wall composition and boost cellulose content, which impact fiber quality (Huang et al., 2016; Chen et al., 2006; Betancur et al., 2010). Sun et al. (2020) found that the cellulose and lignin contents in the stems and roots of transgenic *Arabidopsis* lines were reduced, which revealed that *GhFSN5* is a negative regulator of SCW formation.

In our previous studies, F_2_, F_2:3_, and recombinant inbred line (RIL) (F_2:9_) populations were used for genetic mapping to identify QTLs associated with fiber quality traits in upland cotton. A stable FS QTL on chromosome A06 was consistently identified across multiple generations (Zhang et al., 2020). In this study, we used SLAF-seq combined with bulk segregant analysis (SLAF-BSA-seq) to map a candidate interval for FS for the F_2_ population. We were able to fine map a smaller genomic region, *qFS_A06_*, with which the identified QTL overlapped on chromosome A06. Cloning analysis revealed *GhALDH7B4* as the potential candidate gene for *qFS_A06_*, and this was supported by a nonsense mutation and the correlation of *GhALDH7B4* gene expression with FS. Functional validation experiments using VIGS in cotton and overexpression in *Arabidopsis thaliana* demonstrated that *GhALDH7B4* positively regulates cotton FS.

## Materials and methods

### Plant materials

The parents (Ji567 and Ji228) of the high fiber quality hybrid cotton Jí1518 and its offspring F_2_ and RIL populations were employed in this study. Zhang et al. (2020) presented an extensive overview of the population’s growth process and the phenotypic evaluation of fiber quality. Ji567, the female parent, has a high yield and moderate FS. Ji228, the male parent, has high fiber quality and high FS; it has the genetic background of sea island cotton and carries the chromosomal segments from island cotton (Liu et al., 2009). RIL131 and RIL229 were selected from the RIL population because they have similar genome composition except that RIL 229 harbors the target FS QTL on chromosome A06; these RILs exhibited maintained stability of agronomic traits over the past 5 years (Tang et al., 2023).

### SLAF-BSA-seq

Both parents and two bulks were chosen to perform SLAF-BSA-seq. In the F_2_ population, 26 plants had the highest FS (H-bulk), and 31 plants had the lowest FS (L-bulk) (**Table 1**). Genomic DNA from all plants in each group was combined in equal quantities to produce bulks that had a final purity level of 40 ng/µL. The SLAF library construction followed Sun et al. (2013), with 364- to 414-bp DNA strands prepared by Biomics Technologies Company (Beijing, China) for pair-end sequencing using Illumina High-seq 2500 platform (Illumina, CA, USA). The data has been deposited in the NCBI database with the accession number PRJNA1049971. After sequencing, the clean reads from the four bulks were mapped to the *G. hirsutum* reference genome (Zhang et al., 2015). GATK and Samtools were employed for SNP analysis (Li et al., 2009; McKenna et al., 2010). Polymorphic SNPs between bulks were used for association research. SNP-index correlation by Abe et al. (2012) and Euclidean distance (ED) by Deza et al (2009). were integrated for association mapping.

**Table 1:** FS characters statistics of parents, F_2_ population, and RIL lines.

Parents/Generation	Individuals	Mean value	Full distance	Minimum value	Maximum value	Standard deviation	Variance	Skewness	Kurtosis
M	–	28.86	–	–	–	0.16	–	–	–
P	–	34.34	–	–	–	0.22	–	–	–
F_2_	244	29.36	8.9	24.8	33.7	1.84	3.39	0.18	−0.5
L-bulk	31	26.39	2.3	24.8	27.1	0.56	0.31	−0.79	0.4
H-bulk	26	32.01	2.6	31.1	33.7	0.70	0.49	0.66	−0.19
RIL131	–	26.42	–	–	–	1.35	–	–	–
RIL229	–	36.06	–	–	–	0.63	–	–	–

### Physical location search of SSR markers

The study employed SSR markers previously used for fiber quality QTL mapping in the population (Zhang et al., 2020) and analyzed their physical locations through literature reported (Liu et al., 2016) and the CottonGen database (https://www.cottongen.org/) (Yu et al., 2021).

### Sequence alignment and analysis

Using the PrimeStar HS high-fidelity enzyme (TaKaRa, China), potential genes were amplified from fiber cDNA of the two parents. For each PCR product, a minimum of eight clones from the *pEASY*-Blunt cloning vector (Transgen, China) were sequenced. Sequence alignment was performed using the DNAMAN 8.0, and the protein sequence was subjected to BLAST search in the NCBI database (https://blast.ncbi.nlm.nih.gov/Blast.cgi). Conserved domains of proteins were analyzed by the Pfam database (http://pfam.xfam.org/).

### Verification of the association between FS and a SNP in the candidate gene *GhALDH7B4_A06*

Twenty RILs were arbitrarily selected to investigate the potential association between the *GhALDH7B4_A06* SNP and FS. The SNP was confirmed through Sanger sequencing, and the primers used are listed in **Supplementary Table 1**. The FS phenotype was determined on the basis of the average over the preceding 3 years.

### Gene expression analysis using quantitative real-time PCR (qRT-PCR)

Various tissues (leaf, stem, and root); ovules at 0 days post-anthesis (DPA); and fibers at 5, 10, 15, 20, and 25 DPA were retrieved from healthy plants of both parents, RIL131 and RIL229. Three biological copies were used in this research. RNA extraction, cDNA synthesis, and qRT-PCR were carried out with the RNAprep Pure Plant Kit (TIANGEN, China), the PrimeScript RT Reagent Kit (TaKaRa, China), and TB Green Premix Ex Taq II (TaKaRa, China), respectively, following the manufacturer’s instructions. As internal control, *histone3* (AF024716) and *TUB2* were employed for gene expression analysis in cotton and *Arabidopsis*, respectively. Relative expression rates were assessed using the 2^−ΔΔCt^ method (Livak and Schmittgen, 2001). **Supplementary Table 1** lists the specific primers that were designed using Primer-BLAST (http://www.ncbi.nlm.nih.gov/tools/primer-blast/).

### VIGS of *GhALDH7B4* in cotton

The 300-bp *GhALDH7B4* fragment was cloned into the cotton leaf crumple virus (CLCrV) vector using *Spe*I and *Asc*I restriction enzymes because recent investigations established the virus’s capacity to influence SCW synthesis during cotton fiber development (Liu et al., 2019; Tian et al., 2022). Primers used are shown in **Supplementary Table 1**. *Agrobacterium* strain LBA4404 was used to transform the VIGS vectors. The bacterial liquid involved in the test mainly included the auxiliary plasmid CLCrVB, the empty vector CLCrVA, the positive control vector CLCrVA-PDS, and the target gene vector CLCrVA-*GhALDH7B4*.

The transformed LBA4404 was injected into the RIL229 plants with high FS using established protocols (Gu et al., 2014; Tian et al., 2022). The silencing effect was initially evaluated by the whitening symptoms observed in the cotton plants expressing the positive control *CLCrV : PDS*. To extend the duration of gene silencing and maintain its efficacy during later stages, a secondary injection was administered at the leaf axil of the primary stem in the early phase of cotton squaring.

Gene silencing efficiency was determined by qRT-PCR using 25-DPA fibers of *CLCrV:00* negative control and *CLCrV : GhALDH7B4* plants. The cotton fiber was harvested per plant when the cotton boll opened naturally and matured. Six strains with high silencing efficiency were selected, and fiber samples from two plants were randomly combined as a biological replicate. Each set comprised three biological replicates for the assessment of fiber quality.

### Overexpression of *GhALDH7B4_A06* in *Arabidopsis*

The open reading frame of *GhALDH7B4_A06^O^* was inserted into the pCAMBIA1302 vector with 35S CaMV promoter using homologous recombination and Golden Gate seamless assembly techniques to construct the *GhALDH7B4_A06^O^* overexpression vector. The primers that were used are outlined in **Supplementary Table 1**. *Arabidopsis thaliana* transformation was conducted with the flower dipping method (Qin et al., 2017). Positive plants were screened in a medium of 1/2 Murashige and Skoog (MS) + Hygromycin B (75 mg/L) to the T_3_ generation. Both wild-type (WT) and transgenic *Arabidopsis* plants were subjected to consistent growth conditions and standard maintenance protocols. At maturity, which typically occurred after approximately 8 weeks, the first stem node of flower stems from both transgenic and WT plants, measuring approximately 10 cm in length from the base to the apex, was chosen for evaluation. The cellulose, hemicellulose, and lignin contents of samples were analyzed using specific detection kits (G0715W, G0716W, and G0708W) (Grace, China) designed for plant material.

### Phenotypic detection and statistical analysis

USTER HVI1000 M700 (Uster Technologies, Switzerland) large-capacity cotton detector was used to test the characteristics of cotton fiber quality, including FL, FS, FM, FU, and FE. All experiments were independently repeated a minimum of three times. Data were analyzed with Microsoft Excel 2021 and SigmaPlot 14.0. Significant differences were determined by ANOVA.

## Results

### Fiber qualities of parents, recombinant lines, and populations

**Figure 1:**
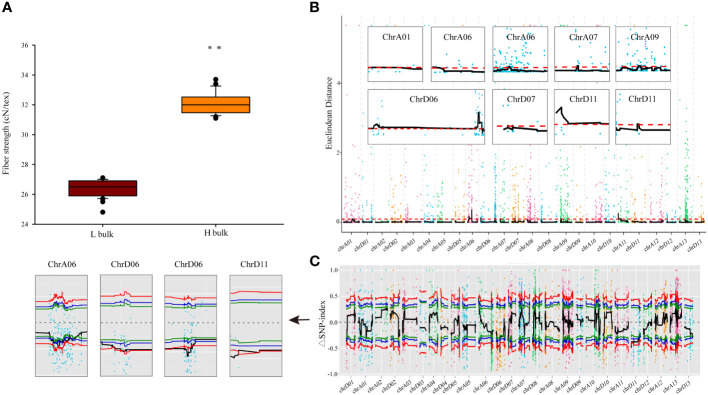
BSA gene bulk and genetic mapping of FS phenotypes. **(A)** FS values in different BSA gene bulks. Error bars represent the SD of different individuals in respective bulks, ** indicates significant difference at p < 0.01. **(B, C)** Statistical algorithms used to map FS candidate genes under default parameters: Euclidean distance association analysis **(B)** and SNP-index association analysis **(C)**. Each colored point represents the calculated ED value or ΔSNP-index value, whereas the black lines indicate the respective fitted values. In the ED algorithm graph **(B)**, red dashed lines indicate thresholds. In the SNP-index algorithm graph **(C)**, the red, blue, and green lines represent thresholds with confidence levels of 0.99, 0.95, and 0.90, respectively.

In this investigation, the parents and offspring of the hybrid Ji1518 were examined. Overall, there were significant variations in FS between the two parents (Ji567, 28.86 cN/tex; Ji228, 34.34 cN/tex), and the F_2_ population consisting of 244 individuals displayed a continuous range of FS variation (**Table 1**). Significant discrepant FS values were observed between the H-bulk (31.1–33.7 cN/tex) and L-bulk (24.8–27.1 cN/tex) selected from the F_2_ population, which can serve as representatives of high and low FS phenotypes (**Table 1**; **Figure 1A**). RIL131 and RIL229 displayed differences in the loci of the FS QTL on chromosome A06, as evidenced by genotyping indicating the presence of a SNP in the coding sequence (CDS) region of the candidate gene *GhALDH7B4_A06*, with RIL131 carrying the *GhALDH7B4_A06^S^* allele and RIL229 carrying the *GhALDH7B4_A06^O^* allele. RIL229 displayed comparable agronomic traits to RIL131 but demonstrated significantly higher FS, with a notable average difference of 9.64 cN/tex (**Table 1**).

### FS QTL identification by SLAF-seq in (Ji567×Ji228) F_2_ population

To identify the major QTL governing cotton FS, SLAF-seq was performed on four libraries from two parents and two bulks from its F_2_ descendants (H bulk and L bulk). A total of 307,849 SLAF tags were obtained (**Supplementary Table 2**), with a uniform distribution across the genome’s chromosomes (**Supplementary Figure 1A**). The average sequencing depth of parents was 25.01× and that of gene bulk with different FS was 34.17× (**Supplementary Table 3**). A total of 125,969 SNPs were uniformly distributed on each chromosome (**Supplementary Figure 1B**). Subsequently, an association analysis was performed using 6,758 high-quality SNPs, which were obtained after rigorous filtering processes (**Supplementary Table 4**).

The ED and SNP-index methodologies were applied to identify QTLs linked to FS. The ED approach, with a threshold of 0.10, revealed that nine QTLs spanning a cumulative length of 26.43 Mb were identified on seven chromosomes (A01, A06, A07, A09, D06, D07, and D11) and encompassed 1,270 genes (**Figure 1B**; **Table 2**). Among these, two QTLs were located on both chromosomes A06 and D11, whereas the rest were single-locus QTLs. Additionally, employing the ΔSNP-index method with a confidence level of 0.99 revealed five QTLs that included 244 genes covering a range of 5.23 Mb on three chromosomes (A06, D06, and D11) (**Figure 1C**; **Table 2**). Integration of results from both methodologies led to the identification of four potential FS-associated QTLs distributed on chromosomes A06, D06, and D11, with a total length of 4.96 Mb and encompassing 242 genes (**Table 2**; **Supplementary Table 5**). Notably, the mapping interval on chromosome D06 exhibited the greatest size, whereas the region on chromosome D11 harbored the highest number of genes, with the smallest mapping interval and least number of genes identified on chromosome A06.

**Table 2:** QTL regions by the two association analysis methods.

Association analysis methods	Chromosome	Start (bp)	End (bp)	Size (Mb)	Gene_Number
Euclidean distance	chrA01	54,443,439	55,480,277	1.04	4
	chrA06	1,464,921	2,617,853	1.15	79
	chrA06	90,575,709	90,898,748	0.32	5
	chrA07	21,370,741	21,506,919	0.14	1
	chrA09	5,527,944	5,809,446	0.28	5
	chrD06	9,146,483	27,491,385	18.34	583
	chrD07	1,580,214	1,580,214	0	1
	chrD11	11,392	4,988,373	4.98	582
	chrD11	18,574,183	18,751,309	0.18	10
	Total	–	–	26.43	1270
SNP_index	chrA06	90,738,206	90,826,117	0.09	2
	chrD06	10,178,496	10,670,314	0.49	24
	chrD06	24,434,721	27,581,372	3.15	72
	chrD06	27,625,118	27,804,679	0.18	1
	chrD11	11,392	1,332,349	1.32	145
	Total	–	–	5.23	244
Combination	chrA06	90,738,206	90,826,117	0.09	2
	chrD06	10,178,496	10,670,314	0.49	24
	chrD06	24,434,721	27,491,385	3.06	71
	chrD11	11,392	1,332,349	1.32	145
	Total	–	–	4.96	242

### Identification of FS-related candidate genes on chromosome A06

**Figure 2:**
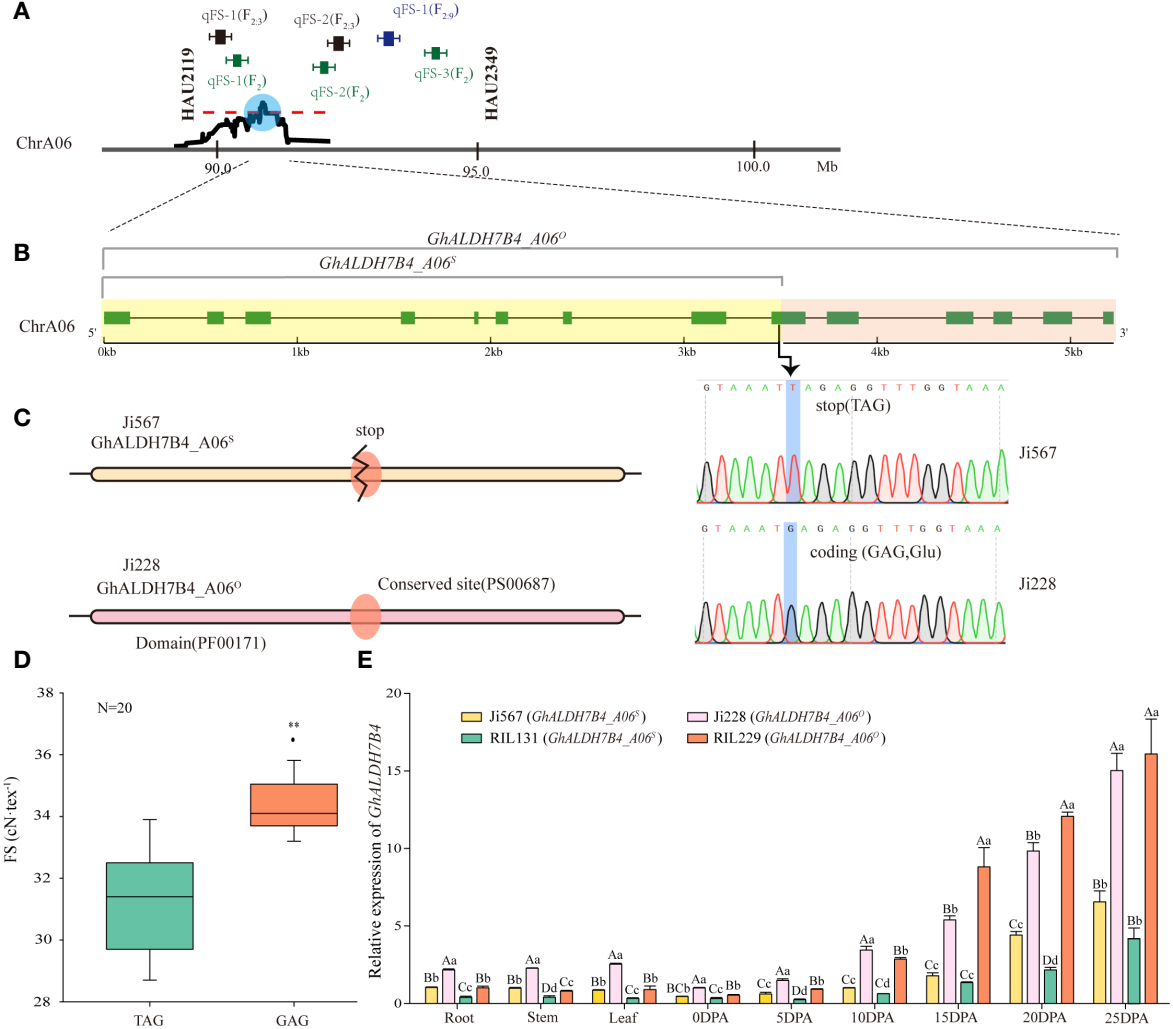
Designation, sequence variations, and differential expression of *GhALDH7B4_A06*. **(A)** QTL interval determination by comparing QTL mapping results based on SSR markers and SLAF-BSA-seq. **(B)** Allelic variations in *GhALDH7B4_A06*. **(C)** Prediction of the protein domain and conserved site of *GhALDH7B4_A06.* ** indicate significant differences at p < 0.01. **(D)** Correlation of FS and allelic variations in *GhALDH7B4_A06* in 20 RILs. **(E)** *GhALDH7B4* expression in different tissues of parents and two RILs. Lowercase and uppercase letters indicate significance at p < 0.05 and p < 0.01, respectively.

To narrow down the target region within the identified extensive interval, integration of previous QTL mapping results for fiber quality traits using SSR markers was conducted. Previous investigations across various populations, including F_2_, F_2:3_, and RIL populations, identified linkage groups associated with FS on chromosome A06, and two SSR markers—HAU2119 and HAU2349—consistently co-segregated within the same linkage group. In the F_2_/F_2:3_ populations, three FS QTLs were positioned between these markers, which explained the phenotypic variances ranging from 3.33% to 13.14%. However, in the RIL population, a single QTL was identified as the exclusive FS QTLs using SSR markers and was associated with 5.10% of the phenotypic variance. We tentatively hypothesize the existence of a stable major-effect QTL between the two markers on chromosome A06 based on the study population. Upon retrieving the physical positions of these markers from the CottonGen database (**Supplementary Table 6**), we identified that the QTL (90.74–90.83 Mb) on chromosome A06 via SLAF-BSA-seq fell within the region demarcated by HAU2119 and HAU2349 (**Figure 2A**), whereas the stability of the presence of QTL on chromosome D06 and D11 by SLAF-BSA-seq cannot be confirmed as no relevant QTL were previously mapped in the RIL population by SSR markers. Therefore, the region on chromosome A06 was selected for further investigation and designated as *qFS_A06_*, and it spanned an 87.9-kb interval.

### Structural and expression analyses of candidate gene *GhALDH7B4 _A06*

A gene located within the chromosome A06 interval was isolated from fiber cDNAs of the parental lines Ji567 and Ji228 and demonstrated a substantial genetic variation between the two lines. In Ji228, the gene’s CDS full length was determined to be 1,527 bp, whereas an SNP variant was identified at position 772 bp in Ji567 that resulted in a G-to-T substitution. This nucleotide change led to the conversion of the normal codon (GAG) to a stop codon (TAG) (**Figure 2B**), which caused premature termination of protein translation.

Protein domain prediction analysis indicated that the presence of stop codons resulted in incomplete structural domains and the absence of conserved glutamic acid active sites (PS00687) (**Figure 2C**). This alteration was discovered to be associated with FS variations in 20 RILs (**Figure 2D**). In particular, the gene did not exhibit any nonsense mutations in the RILs with FS exceeding 34 cN/tex. Therefore, the gene could be divided into full-length and truncated types in the study population. We tentatively assigned this gene as a candidate for *qFS_A06_*.

The candidate gene encoded a protein consisting of 508 amino acids with aldehyde dehydrogenase activity and exhibited 84.84% similarity with AtALDH7B4; thus, it was named *GhALDH7B4* in cotton. This gene had one copy in each of the At and Dt sub-genomes in upland cotton, with 17 nucleotide differences in the CDS region, which led to the nomenclature of *GhALDH7B4_A06* and *GhALDH7B4_D06* (GenBank: PP584503) based on their chromosomal locations. The full-length variant on chromosome A06 was named *GhALDH7B4_A06^O^* (GenBank: PP210923), whereas the truncated form was named *GhALDH7B4_A06^s^* (GenBank: PP584502) (**Supplementary Table 7**). Cloning results indicated no differences in the *GhALDH7B4_D06* CDS between the two parents (**Supplementary Table 7**).

qRT-PCR was employed to assess the expression pattern of *GhALDH7B4* (**Figure 2E**). The consistent predominance of *GhALDH7B4* expression in cotton fibers during 15–25 DPA, irrespective of the sample source, indicated its potential involvement in SCW formation; therefore, it likely contributes to FS development in cotton. Furthermore, the parent Ji228 and its progeny RIL229, which carry *GhALDH7B4_A06^O^*, displayed significantly higher or extremely significantly higher expression levels across diverse tissues compared with Ji567 and RIL131, which carry *GhALDH7B4_A06^S^*, indicated a possible association between gene expression levels and genotypes.

### Functional analysis of *GhALDH7B4 _A06* by silencing in cotton and overexpression in *Arabidopsis*

**Figure 3:**
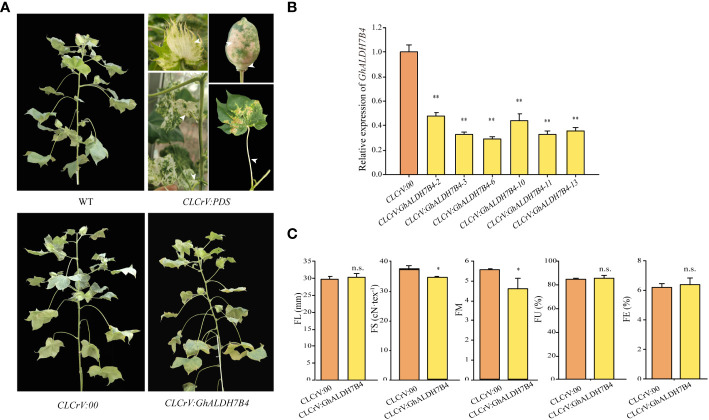
Phenotype, gene expression in fiber, and fiber quality traits of *GhALDH7B4*-silenced cotton plants. **(A)** Phenotypic characteristics in flowering and boll stages after the VIGS injection. **(B)** Gene expression analysis of *GhALDH7B4* by qRT-PCR. **(C)** Fiber quality trait analysis. Error bars represent the SD of three replicates. * and ** indicate significant differences at p < 0.05 and p < 0.01, respectively, and n.s. indicates not significant.

A VIGS experiment was performed in cotton to confirm the role of *GhALDH7B4_A06* in FS formation. The findings demonstrated the persistence of whitening symptoms in various tissues, including cotton boll bracts, boll shells, and leaves, throughout boll development in the positive control group, which indicated successful silencing (**Figure 3A**). Notably, *CLCrV : GhALDH7B4* plants exhibited a slender and fragile phenotype, although no discernible changes in external morphology were observed post-injection compared with the WT plants (**Figure 3A**). qRT-PCR analysis displayed a silencing efficiency of 65.16% for *GhALDH7B4*, with values ranging from 52.91% to 70.18% (**Figure 3B**). Fiber quality test results indicated a significant reduction in FS and FM in *CLCrV : GhALDH7B4* cotton fiber, with reductions of 2.83cN/tex and 0.97, respectively. However, no substantial differences were observed in other fiber quality parameters compared with the negative control (**Figure 3C**). This VIGS experiment provided compelling evidence that supported the potential positive role of *GhALDH7B4* in cotton FS development.

**Figure 4:**
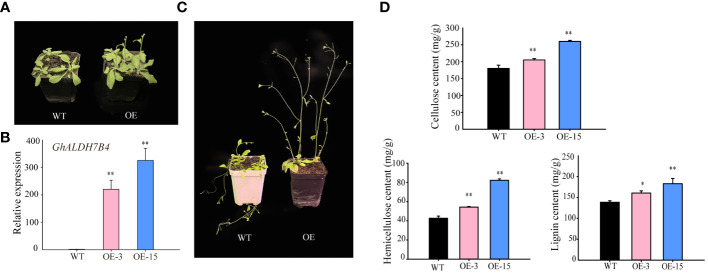
*GhALDH7B4_A06* overexpression in *Arabidopsis.* **(A)** Phenotypic characteristics in 3-week-old *Arabidopsis* plants. **(B)** Phenotypic characteristics in 5-week-old *Arabidopsis* plants. **(C)** Gene expression analysis of *GhALDH7B4* by qRT-PCR. **(D)** Assay of cell wall component (cellulose, hemicellulose, and lignin) contents from the stem in *GhALDH7B4_A06* transgenic *Arabidopsis*. Cell wall component contents were determined in *Arabidopsis* stems older than 8 weeks of age. Error bars represent the SD of three replicates. * and ** indicate significant differences at p < 0.05 and p < 0.01, respectively. WT, wild type; OE3 and OE15, *GhALDH7B4 _A06* transgenic lines.

For complementary functional assessment, we executed an overexpression experiment of *GhALDH7B4_A06^O^* in *Arabidopsis* to investigate its potential regulatory role in SCW biosynthesis. Phenotypic evaluation of the transgenic lines revealed accelerated growth rates (**Figure 4A**) and enhanced stem strength compared with the WT (**Figure 4B**). Subsequent evaluation focused on two transgenic lines (OE-3 and OE-15) characterized by elevated expression levels of *GhALDH7B4_A06^O^* (**Figure 4C**). Analysis of cell wall constituents indicated higher levels of cellulose, hemicellulose, and lignin in the transgenic progenies relative to the WT (**Figure 4D**). In particular, the most prominent disparity in cellulose content was observed in the overexpression lines compared with the WT, which demonstrated that *GhALDH7B4_A06^O^* overexpression may stimulate augmentation of compounds associated with SCW synthesis.

## Discussion

### *GhALDH7B4_A06* is a candidate gene associated with cotton FS

The combination of BSA and sequencing is effective for rapidly mapping major QTLs and is widely used in various crops (Takagi et al., 2013; Illa-Berenguer et al., 2015; Chen et al., 2021). In cotton, this approach has been applied to studies on important traits such as fiber quality, boll weight, agronomic traits, and disease resistance (Zhang et al., 2016; Cui et al., 2021; Ma et al., 2022; Jia et al., 2023; Zhang et al., 2024). In our study, an F_2_ population was used for SLAF-BSA-seq, and four QTLs associated with FS were mapped to three chromosomes. The large interval encompassed 242 genes. However, we focused on the small mapping region *qFS_A06_*, primarily because FS-related QTLs were detected within the linked region of chromosome A06 across the F_2_, F_2:3_, and RIL populations using SSR markers. As *qFS_A06_* resides within this region, we hypothesize that it represents a stable major-effect QTL within these populations. Through gene cloning analysis in the parents, one gene, *GhALDH7B4_A06*, was identified within *qFS_A06_*, and it had a critical nonsense mutation within the CDS region. Subsequent genotyping of RILs and gene expression analysis confirmed the stable presence of this variant locus, which showed significant correlation with FS in these populations.

Liu et al. (2016) employed 6,975 F_2_ populations to map QTL clusters associated with FL, FS, FM, and FU on chromosome A06 of upland cotton. The QTL identified in their investigation overlapped with *qFS_A06_* in our research, and *GhALDH7B4_A06* was also considered one of candidate genes. Moreover, Liu et al. (2023) identified *GhALDH7B4_A06* as a candidate gene related to SCW biosynthesis through comparative transcriptome analysis of fiber tissues between *Gossypium barbadense* and *G. hirsutum*. However, neither of those studies reported on the presence of site mutations in this gene. Our findings revealed that a nonsense mutation in *GhALDH7B4_A06* determined the functional expression of the protein. On the basis of the expression of this gene reported in the above studies, we speculate that this site mutation may be prevalent in upland cotton because one parent, Ji228, is the genetic background of sea island cotton and has chromosomal segments from island cotton (Liu et al., 2009). The full-length–type protein *GhALDH7B4_A06^O^* showed 100% similarity in sequence alignment with the gene on chromosome A06 of *G. barbadense* (GenBank: KAB2078339.1). Such high similarity was not found in proteins from upland cotton, which indicates that the full-length *GhALDH7B4_A06^O^* likely originated from high fiber quality island cotton. In a future study, we will investigate the germplasm of upland cotton from different sources to test our hypothesis that *qFS_A06_* represents a stable major-effect QTL. Furthermore, FS is a quantitative trait controlled by multiple genes (Zhang et al., 2017). Because of the broad mapping intervals, we will perform detailed fine mapping studies in the future to pinpoint candidate genes for the other candidate regions identified by SLAF-BSA-seq in this study.

### *GhALDH7B4_A06* positively regulated FS in upland cotton

FS primarily develops during the SCW thickening stage (Zang et al., 2022). On the basis of identified genes with regulatory functions during this period in cotton, Wen et al. (2023) constructed a model to enhance our understanding of the regulatory mechanisms of genes expressed during SCW development. However, there is still no comprehensive understanding of the intricate regulatory network, which necessitates further identification of FS-related genes.

*GhALDH7B4* encodes an aldehyde dehydrogenase and is the sole member of the ALDH7 family in upland cotton, with one copy present in each of the At and Dt sub-genomes (Dong et al., 2017; Guo et al., 2017). *ALDH7B4* has been commonly documented in plants to operate under abiotic stress conditions, including drought, abscisic acid, and salinity (Hou et al., 2022), with its role in cotton fiber development remaining largely unexplored. Our experiments involving gene silencing of *GhALDH7B4_A06^O^* in cotton and overexpression in *Arabidopsis* revealed that *GhALDH7B4_A06^O^* may positively regulate upland cotton FS.

A multitude of *ALDH*s have been identified in various crops such as *A. thaliana*, poplar, and maize, and it was proposed that they likely participate in SCW synthesis (Nair et al., 2004; Guillaumie et al., 2008; Bosch et al., 2011; Tian et al., 2015). It was suggested that *ALDH*s may have distinct roles in different stages of cell wall biosynthesis (Islam and Ghosh, 2022). However, the molecular regulatory mechanisms of *ALDH* genes remain unclear.

As previously mentioned, FS is a quantitative trait controlled by a complex network of multiple genes (Zhang et al., 2017). *GhALDH7B4_A06* is unlikely to act independently but rather exists within this network of interactions. However, the role of *GhALDH7B4_A06* in the regulatory network and its upstream substrate and downstream products remain unknown. Future investigations using transgenic lines for overexpression and gene knockout are anticipated to provide further insight into its function and metabolic pathways and to facilitate its practical application through the development of linked marker combinations.

## Conclusion

This study employed forward genetics using SLAF-BSA-seq and SSR markers to locate a stable FS locus, *qFS_A06_*, and identified the candidate gene *GhALDH7B4_A06*. A premature stop codon within the candidate gene was associated with FS lines of RIL populations and resulted in a truncated variant, *GhALDH7B4_A06^S^*, which may lead to functional protein loss. The subsequent VIGS and overexpression experiments demonstrated that *GhALDH7B4_A06* likely plays a positive regulatory role in FS of upland cotton. This discovery significantly contributes to advancing our understanding of the regulatory network associated with cotton fiber development.

## Data availability statement

The datasets presented in this study can be found in online repositories. The names of the repository/repositories and accession number(s) can be found in the article/**Supplementary Material**.

## Author contributions

LT: Funding acquisition, Investigation, Writing – review & editing, Data curation, Software, Validation, Writing – original draft. CL: Investigation, Writing – review & editing, Formal analysis, Supervision. XL: Investigation, Writing – review & editing, Software. HW: Investigation, Writing – review & editing, Project administration. SZ: Writing – review & editing, Data curation, Software, Validation. XC: Software, Writing – review & editing, Formal analysis, Methodology, Supervision. JZ: Writing – review & editing, Funding acquisition, Investigation.

## Supplementary material

Supplementary Figure 1 SLAF and SNP distribution on genome.
