# Supplementary figures and images for: An aldehyde dehydrogenase gene, GhALDH7B4_A06, positively regulates fiber strength in upland cotton (Gossypium hirsutum L.)
Source: Front Plant Sci. 2024 Apr 26;15:1377682. doi: 10.3389/fpls.2024.1377682 (PMC11082362; doi:10.3389/fpls.2024.1377682)

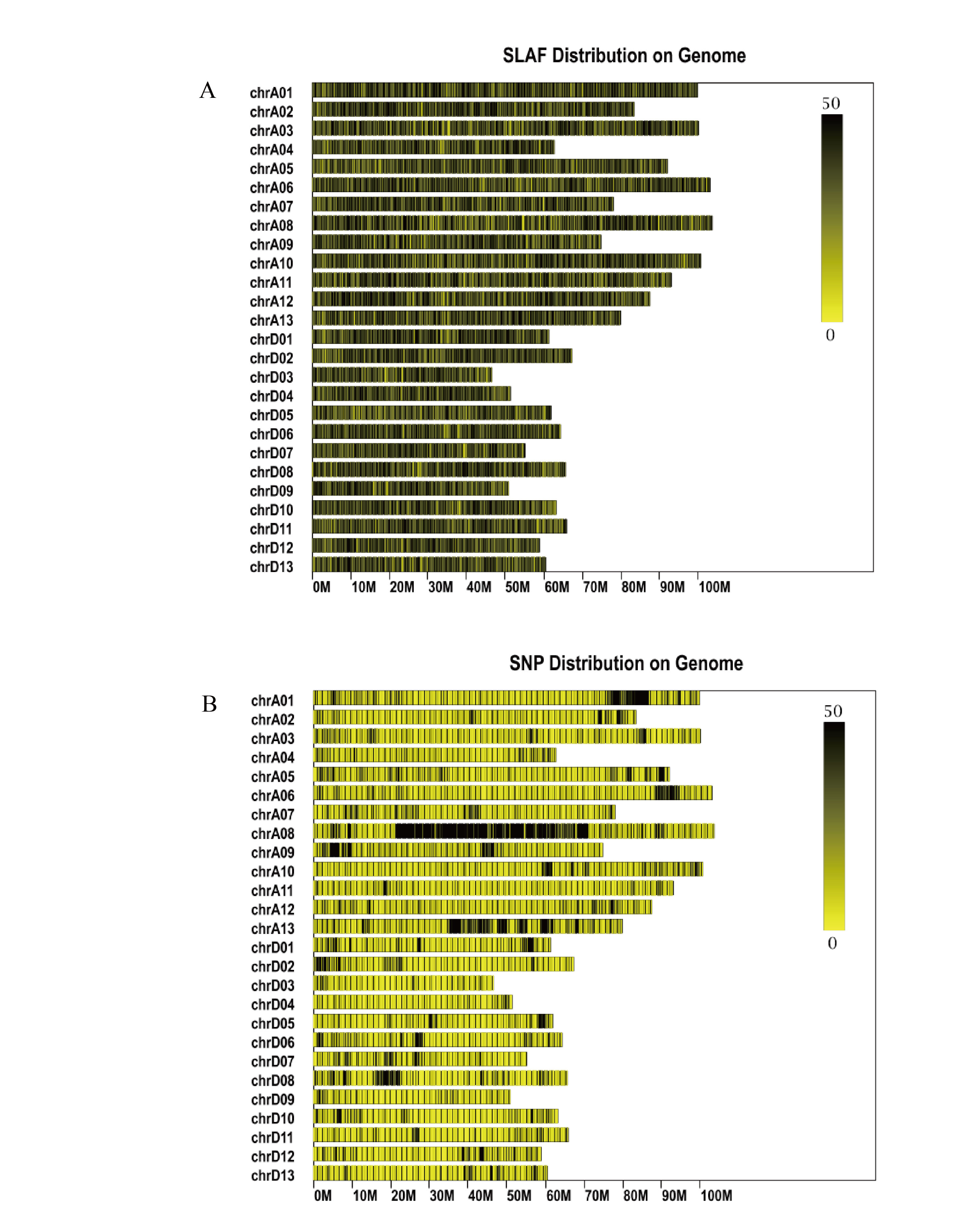

Supplement: Supplementary Figure 1 — SLAF and SNP distribution on genome. [file Image_1.jpg]
